# Ensembl 2019

**DOI:** 10.1093/nar/gky1113

**Published:** 2018-11-08

**Authors:** Fiona Cunningham, Premanand Achuthan, Wasiu Akanni, James Allen, M Ridwan Amode, Irina M Armean, Ruth Bennett, Jyothish Bhai, Konstantinos Billis, Sanjay Boddu, Carla Cummins, Claire Davidson, Kamalkumar Jayantilal Dodiya, Astrid Gall, Carlos García Girón, Laurent Gil, Tiago Grego, Leanne Haggerty, Erin Haskell, Thibaut Hourlier, Osagie G Izuogu, Sophie H Janacek, Thomas Juettemann, Mike Kay, Matthew R Laird, Ilias Lavidas, Zhicheng Liu, Jane E Loveland, José C Marugán, Thomas Maurel, Aoife C McMahon, Benjamin Moore, Joannella Morales, Jonathan M Mudge, Michael Nuhn, Denye Ogeh, Anne Parker, Andrew Parton, Mateus Patricio, Ahamed Imran Abdul Salam, Bianca M Schmitt, Helen Schuilenburg, Dan Sheppard, Helen Sparrow, Eloise Stapleton, Marek Szuba, Kieron Taylor, Glen Threadgold, Anja Thormann, Alessandro Vullo, Brandon Walts, Andrea Winterbottom, Amonida Zadissa, Marc Chakiachvili, Adam Frankish, Sarah E Hunt, Myrto Kostadima, Nick Langridge, Fergal J Martin, Matthieu Muffato, Emily Perry, Magali Ruffier, Daniel M Staines, Stephen J Trevanion, Bronwen L Aken, Andrew D Yates, Daniel R Zerbino, Paul Flicek

**Affiliations:** European Molecular Biology Laboratory, European Bioinformatics Institute, Wellcome Genome Campus, Hinxton, Cambridge CB10 1SD, UK

## Abstract

The Ensembl project (https://www.ensembl.org) makes key genomic data sets available to the entire scientific community without restrictions. Ensembl seeks to be a fundamental resource driving scientific progress by creating, maintaining and updating reference genome annotation and comparative genomics resources. This year we describe our new and expanded gene, variant and comparative annotation capabilities, which led to a 50% increase in the number of vertebrate genomes we support. We have also doubled the number of available human variants and added regulatory regions for many mouse cell types and developmental stages. Our data sets and tools are available via the Ensembl website as well as a through a RESTful webservice, Perl application programming interface and as data files for download.

## INTRODUCTION

Ensembl enables genome science by systematically integrating, harmonizing and presenting data in a consistent manner, both via a web interface (https://www.ensembl.org) and via application programming interfaces (APIs) (1–3). Four times per year we update our data, website, APIs and tools with the latest genomic data and new features. We import primary data, such as assemblies and discovered variants, and then annotate genes and transcripts (4), variants (5), regulatory regions (6) and comparative genomics features (7). These are made freely available, without restriction, to all.

Over the last year, the successful redesign of Ensembl's gene annotation methodology has enabled us to dramatically increase the number of chordate genomes we support. For example, we annotated hagfish (*Eptatretus burgeri*), which is an extreme evolutionary outlier, and many ray-finned fish that are interesting due to their phenotypic diversity. Our gene annotation methods employ a mix of strategies to compute the best possible annotation based on available evidence including extensive multi-tissue transcriptomic data (4). To classify our genes for homology analysis across all phyla, we have developed a new approach using profile hidden Markov models (HMMs). Finally, we have doubled the number of whole-genome pairwise alignments over the last year. Ambitious sequencing efforts, such as the Vertebrate Genomes Project (VGP; https://vertebrategenomesproject.org/), Genome10K (8), Bird 10K (9), Bat 1K (10) and eventually the Earth BioGenome Project (11), are planning to create an ever larger collection of sequenced genomes. As active members of Genome10K, VGP and the Earth BioGenome Project, we plan to incorporate genomes arising from these and other projects into Ensembl using our newly improved pipelines. Delivering scientific insight and creating valuable community resources from these data will drive many of our future developments.

With each new genome published or sequencing technology introduced, opportunities expand for exploring genome evolution, regulation and variation. Alongside supporting an increasing number of species and associated data, we also seek to improve genome interpretation by enriching the genome annotation we produce and provide. Amongst many recent updates, we have doubled the number of variants in human, generated a new primate multiple sequence alignment, and integrated data from a large-scale systematic study of chromatin state in the developing mouse.

Software tools and technologies that facilitate data access and exploration must also change in response to the growth in data size and complexity. To improve interactive analysis, we have focused on the usability of key tools, which are now available as Docker containers. In addition, user data uploads are now supported in more formats, and linkage disequilibrium (LD) is easier to calculate and use via a new tool with both web and command line interfaces.

Our overall goal is to create reference datasets and tools that improve the ability to interpret and understand genomes across the tree of life. We have expanded our resources from 100 annotated assemblies in Ensembl release 90 (August 2017) to 153 annotated assemblies in release 94 (October 2018). Thus, we have seen a 50% growth in the number of supported genomes in just the last 12 months and this growth is in addition to updated annotation on existing assemblies. We believe the expansion in the past year represents a significant step towards our plan to create and distribute annotation for all vertebrate species.

## SUPPORTING ANNOTATION OF ALL SPECIES

### Large-scale annotation of diverse vertebrate species

The efforts currently underway to sequence a broad range of species are expected to create vast quantities of high-quality vertebrate assemblies suitable for integration into Ensembl. Our efforts to re-imagine large-scale genome annotation have come to fruition as, over the past year, we have begun to annotate assemblies with genes and transcripts at a rate not previously possible.

In Ensembl release 91 (December 2017), we annotated twelve new primate species including the crab-eating macaque, pig-tailed macaque, capuchin and the night monkey, and released new annotation on updated assemblies for a further six (gorilla, gibbon, mouse lemur, chimp, tarsier, and baboon). In addition, we updated the Ensembl/GENCODE annotation for mouse and annotated the cat assembly (version 8.0). For Ensembl releases 92 (April 2018) and 93 (July 2018), we annotated a mix of species including goat, marmoset, cat (updated to version 9.0), leopard, tiger, hagfish, mouse and human. Hagfish in particular was challenging due to the extreme evolutionary distance to other vertebrates (12). A combination of robust RNA-seq data and changing alignment parameters to maximize sensitivity led to a hagfish annotation with 16 513 protein-coding genes with 29 049 transcripts.

In Ensembl release 94, in addition to updating human, mouse and mouse lemur, we focused on the annotation of ray-finned fishes, which account for nearly half of all extant vertebrates and exhibit a high level of phenotypic diversity. We annotated 41 new assemblies consisting of four existing species (medaka Hd-rR, fugu, platyfish and cave fish), two strains (medaka HNI and medaka HSOK) and 35 new species (including pike, zig-zag eel, Indian medaka, catfish, elephant fish and a collection of six cichlids). The evidence we used to annotate these fish included available RNA-seq data, mapping of high-quality annotation from zebrafish and alignments of UniProt vertebrate proteins with associated experimental evidence (13).

Across the 41 fish assemblies we annotated a total of 993 666 genes comprising 1 455 312 transcripts in our primary gene sets. For species with RNA-seq data, we generated sample-specific RNA-seq gene tracks in addition to generating the main gene set. Samples include tissues, development stages and environmental conditions. These additional gene tracks indicate which genes are expressed in each sample as well as the dominant transcript structure present at each locus.

To provide links to gene annotation in other databases and resources, we streamlined our external references mapping to enable more frequent updates. While previously we only built links when a gene set was modified, we now update cross-references for all species in every release. This ensures our links to external databases, including RefSeq (14) and UniProt, are current.

To support the new gene annotation, we have also refreshed microarray probe mappings for human, mouse strains, marmoset, cat, goat, *C. elegans*, zebrafish, Ma's night monkey, capuchin, sooty mangabey, gorilla, crab-eating macaque, pig-tailed macaque, mouse lemur, bonobo, chimpanzee, olive baboon, black snub-nosed monkey, golden snub-nosed monkey, Bolivian squirrel monkey, upper Galilee mountains blind mole rat, and guinea pig. New microarray designs have been added for several species.

### Large-scale comparative annotation across vertebrate species

Our whole-genome alignments have been updated to include the new and existing assemblies: Ensembl release 94 now features a 48-way fish multiple alignment, which replaced an 11-way fish alignment introduced in Ensembl release 91 (December 2017). We also introduced a new 24-way primate whole-genome multiple alignment to give greater insights into primate evolution. As we systematically compute whole-genome pairwise alignments against reference species—including human against all vertebrates as well as zebrafish and medaka against all fish—the total number of pairwise alignments we provide has more than doubled in the past year to over 300.

We now use HMMs to classify protein-coding genes into families for our homology analyses including phylogenetic trees, orthologues and paralogues. These developments allowed us to abandon the expensive all-vs-all approach to homology annotation we used for nearly a decade, and will enable us to annotate even more genomes in the future. We compared the HMM-based method with the previous method by analysing the pairwise 1-to-1 orthologue relationships within the gene trees. The move to the HMM-based approach resulted in an approximate 5% change to the annotations compared to our previous method, which exhibited on average a 2% change in results from release to release. Importantly, however, we predict the 1-to-1 relationships will have improved stability across releases. Thus, we expect the percentage release to release change to be significantly smaller in the future. Additional method details are available at http://www.ensembl.org/info/genome/compara/homology_method.html.

Ensembl Genomes (15) are also migrating to the same set of HMMs to define their protein families. Thus, the same family identifiers will be shared between both resources, extending the phylogenetic analyses beyond vertebrates to all eukaryotes.

### Improved data management and availability

As the number of species with publicly available variant data has grown, we have focused on developing a new streamlined method for extracting genotype data from the primary variant archives. Specifically, we improved the integration of Ensembl's variation data (5) with the European Variant Archive (EVA; https://www.ebi.ac.uk/eva/) by configuring the Ensembl website to use EVA genotype data directly using Tabix indexed VCF files (16). Doing so enables us to integrate and release more comprehensive genotype and variant frequency data via Ensembl, without the need to copy these data directly into our infrastructure (3). Data for horse and dog are currently available using this approach.

We have also developed a prototype RESTful API at http://test-metadata.ensembl.org/ to aid in finding available resources in Ensembl. This service, which facilitates discovery and retrieval of specific datasets, is updated every release with the latest datasets and reports both what species and assembly versions are available as well as what data types (e.g. variation, regulation, etc.).

## GENOME INTERPRETATION

### New and expanded annotation resources

Over the last year, the number of short variants in our human database has doubled to over 600 million. The majority of these additional variants were discovered in the Genome Aggregation Database (gnomAD) (17) or Trans-Omics for Precision Medicine (TOPMed) projects, which sequenced the exomes of over 123 000 individuals and whole genomes of over 15 000 individuals (gnomAD), and the whole genomes of over 62 000 individuals (TOPMed), respectively. These projects provide an extensive catalogue of rare variants. Frequency distributions can be viewed in Ensembl for the full sample sets for both projects and for the seven gnomAD population groupings. To further aid variant interpretation, our variant pages now have links to PharmGKB (18), which has curated data on the influence of variants on drug response.

New this year is a variation database for goat (19), with over 37 million variants. Goat is a species with worldwide importance as a source of food and milk, but also skin and hair. Our browser displays Ensembl Variant Effect Predictor (VEP) (20) annotation for these variants using the gene annotation described above and SIFT predictions (21) to help identify potentially damaging protein changes. Genotype and allele frequency data are available for 195 individuals from the NextGen project (22). Variants from the Illumina_GoatSNP50 genotyping array can also be viewed as a track alongside gene and other annotation in our ‘Region in Detail’ views.

We used a large-scale and systematic epigenetic study on mouse embryogenesis (bioRxiv: https://doi.org/10.1101/166652) to enhance our annotation of regulatory elements in Ensembl. These data were integrated into the mouse version of the Ensembl Regulatory Build (23), which increased our coverage from 8 to 79 cell types and tissues, many of which are mapped to specific developmental stages and therefore create an important dataset to track epigenetic modifications during development. As a result, the number of annotated regulatory elements, including enhancers, transcriptional regulators and chromatin state, went up substantially from 313 665 to 419 000 and now covers ∼15% of the mouse genome.

**Figure 1.**
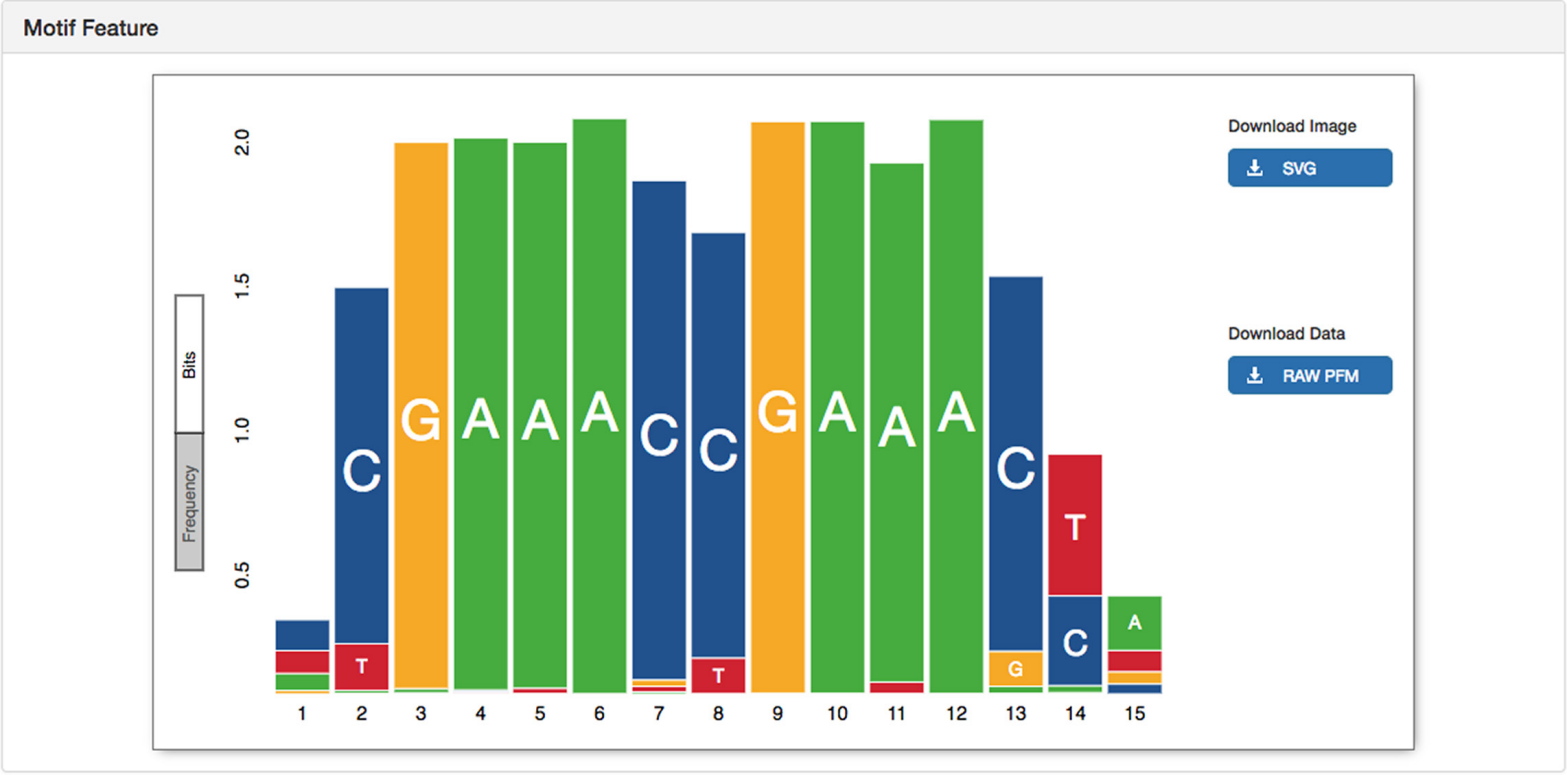
Example of Ensembl's new motif feature visualization, displaying the position weight motif of IRF4, IRF5, IRF8 and IRF9, using height to represent information content.

We have improved our characterization of transcription factor binding specificities by importing 632 human and 85 mouse transcription factor binding motifs imputed through SELEX (24–26) and mapping them onto the respective genomes. These potential binding sites were then compared to available epigenomic datasets to highlight those with known occupancy. This new collection greatly expands our repertoire of known motifs and covers a significant fraction of all known transcription factors (27). Furthermore, we developed a new visualization for the sequence logos of regulatory motifs, which user testing demonstrated to be a simpler and more accurate representation of the data (see Figure 1). Rather than the commonly-used stretched base visualization with coloured letters, this uses solid blocks of colour to represent the information content at each base. The new display scales well, both horizontally and vertically, without losing legibility. Data can be downloaded from the image and the image itself can be exported in SVG format to enable reuse and integration into publications and presentations. The visualization is accessible through clicking on a motif feature on any location-based display.

Structured metadata, in parallel to the genomic annotation we create, is vital to enable large-scale genome interpretation. For Ensembl's gene annotation in all species, we add metadata to describe the type of gene (‘biotype’) because, for example, when diving into the annotation of a single transcript, it matters whether it is a small nucleolar RNA (snoRNA) or a small nuclear RNA (snRNA). In large-scale analyses however, it can be more relevant to include or exclude all non-coding RNAs, without the need for the distinction between different subtypes. To cater for both aspects of this problem, we combined close to a hundred different biotypes into three logical and practical groups useful for research: coding, non-coding and pseudogenes. These groups can be queried through our REST API (2), for example https://rest.ensembl.org/info/biotypes/groups/coding?content-type=application/json and are available in our GFF3 files (ftp://ftp.ensembl.org/pub/current_gff3).

### Genome interpretation tools

We have improved access to our LD calculations that were previously only available via the Perl and REST APIs. We added both a simple web tool and a highly configurable command line tool for more in depth analysis. The web tool returns population-specific LD results by region, single variant, or variant list for human, sheep and goat populations up to a region size of 500 kb. The command line tool can be configured to use any appropriate genotype data in VCF files, where the variants are stored in our databases. The advantage of the command line tool is that it can process much larger regions limited only by the memory available on the machine, thus making it useful for fine-mapping studies and other analyses.

To empower analysis of variant data in any region of the genome, we implemented a new region-specific variant table this year. For each variant, information such as global minor allele frequency, supporting evidence, clinical significance, phenotype associations and most severe predicted consequence can be displayed. The table can be filtered by these different attributes and also be exported.

**Figure 2.**
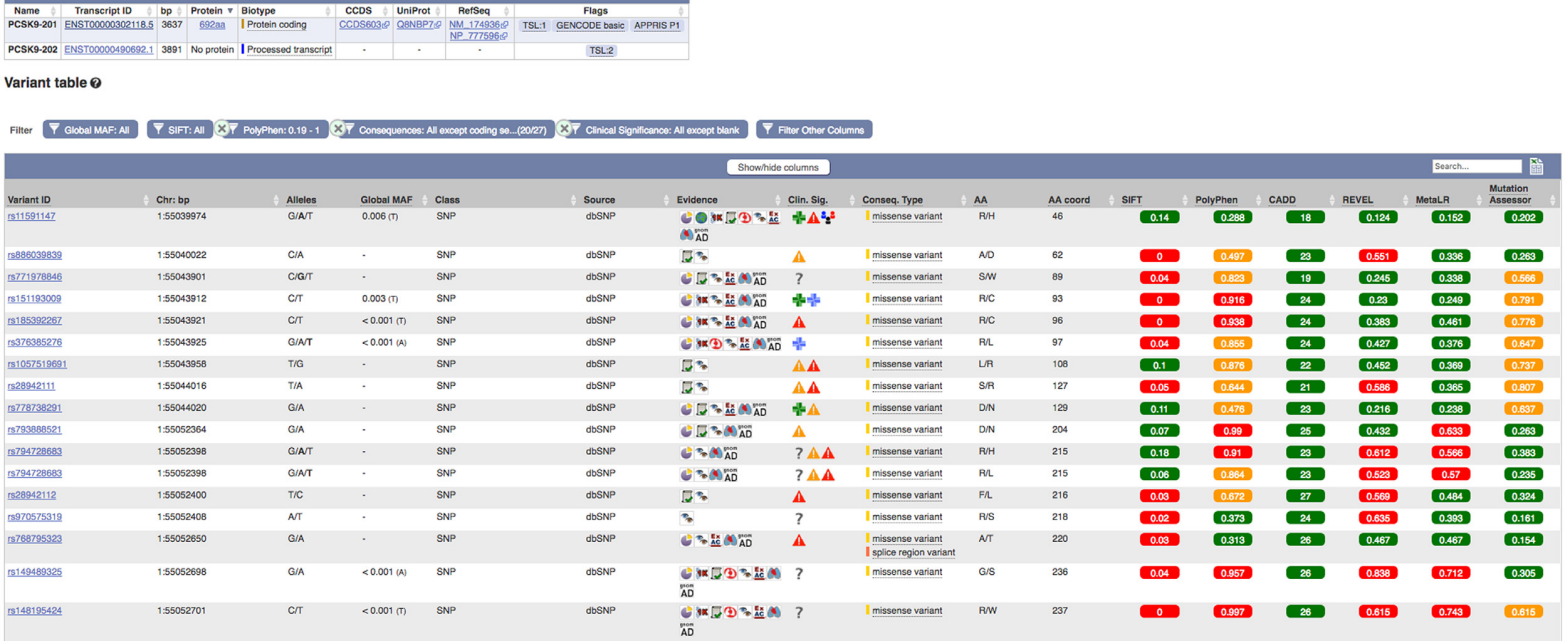
The variant table for a transcript summarizes the annotation across the transcript for each variant, including the global allele frequency, clinical significance, consequence, allele change as well as five different prediction algorithms to assess the variant impact: SIFT (21), PolyPhen-2 (33), CADD (30), REVEL (29), MetaLR (31) and MutationAssessor (32).

As no algorithm is optimal in all situations, the Ensembl VEP tool incorporates an extensive list of prediction algorithms to support evaluation of the potential deleteriousness of a variant. This year we developed new VEP ‘plugins’ to expand this functionality including Missense Tolerance Ratio (MTR) (28) and the Rare Exome Variant Ensemble Learner (REVEL) (29). MTR provides scores to quantify purifying selection for a given window of the coding genome using frequency data from ExAC (17). Allele frequency, as reported in VEP, is commonly used as a first pass to filter for pathogenic variants. REVEL combines predictions from 13 tools and 18 scores to predict the pathogenicity of missense variants and to distinguish pathogenic from rare neutral variants (i.e. those with allele frequencies <0.5%). We have also extended the range of impact predictions available in our transcript variant tables, adding results from CADD (30), MetaLR (31), MutationAssessor (32) and REVEL, alongside the pre-existing SIFT and PolyPhen-2 results (33) (see Figure 2). For consistency with other resources, we use pre-calculated results from the CADD and dbNSFP (34) resources for these additional scores.

## RESEARCHER-DRIVEN ANALYSIS

This year, we released Docker containers for a number of our tools to simplify installation and update procedures for these tools on any platform. These are available from our Docker Hub portal (https://hub.docker.com/u/ensemblorg/) and include the Ensembl VEP and eHive (35).

We have implemented a new REST endpoint which reports phenotype associations for a gene. Our population endpoint has been updated to list the individuals in the population and our variant endpoint now returns the list of genotyping chips which contain assays for the variant. Our Ensembl VEP REST service has been updated to accept a location and alternate allele as minimum input to ease usability. The representation of allele frequency data used in our VEP endpoints has also been improved for clarity.

Furthermore, our Perl API for describing epigenomic datasets has been clarified to make it easier to learn and use. Terms such as ResultSet, InputSubset and AnnotatedFeature have been replaced by more common terms such as Alignment, ReadFile and Peak.

We have embedded the interactive pathway widget from Reactome (36) into the Ensembl browser to help understand the biological context of the products of genes. This visualization shows the latest data available in Reactome. The display is accessible in Ensembl by clicking on the ‘Pathway’ link in the left hand navigation menu (e.g. https://www.ensembl.org/Homo_sapiens/Gene/Pathway?g=ENSG00000139618;r=13:32315474–32400266).

We have also made a number of improvements to support data upload to Ensembl. We added support for two additional file formats: bigPsl for alignments (37) (https://genome.ucsc.edu/goldenpath/help/bigPsl.html) and interact for genome interactions (https://genome.ucsc.edu/goldenpath/help/interact.html). We made interpretation of wiggle data easier by allowing the scale to be manually set.

We continue to support the Ensembl BioMart (38) installation to facilitate flexible data queries and interface to the Bioconductor platform (39).

Finally, TrackHubs (40) or other remote files that have been previously attached can now be disconnected and later reconnected from the display. This results in a faster browsing experience with easy access to the data when required because they are not automatically loaded.

## OUTREACH

Ensembl continues to offer training courses globally and can travel to institutes all over the world to deliver training on using the Ensembl browser and REST APIs (https://training.ensembl.org/hosting). Our REST API courses are also available as Jupyter Notebooks (41) and available online from Microsoft Azure Notebooks (https://notebooks.azure.com/ensembl-training). We offer Train the Trainer courses, which empower local trainers to deliver future Ensembl training. For those unable to attend our training courses, we have a number of short training videos available on our YouTube channel (https://www.youtube.com/EnsemblHelpdesk) with longer structured training available on the EMBL-EBI Train Online Platform (https://www.ebi.ac.uk/training/online/).

## FUTURE WORK

Our strategy for the future will focus on the three goals described here: to support all vertebrate species, to facilitate genome interpretation and to distribute genomic data in a manner that enables researcher-driver analysis. In the near term, we are collaborating with RefSeq to jointly improve our annotation of human protein-coding transcripts and converge on a subset of transcripts that are identical from 5’UTR to 3’UTR. We plan to improve the availability and visualization of protein annotations for variants both in the browser views and in the Ensembl VEP. Specifically, we are developing a new view to show the location of protein-disrupting variants on three-dimensional protein structures with protein domains and exons highlighted. To advance analysis of non-coding genomic regions, we will be incorporating a broad collection of experiments across a wide number of cell types, cell lines, tissues and species into the Ensembl Regulatory Build through our participation in both the International Human Epigenome Consortium (IHEC) (42) and the Functional Annotation of Animal Genomes (FAANG) consortium (43).

Finally, we are in the process of completely redesigning the Ensembl website. Our reimplementation will feature more client-side data processing and rendering to create a more immersive and responsive experience. We will release public alpha and beta versions of the site over the course of 2019 as we transition from our old infrastructure to the new one. During the same period, we will be conducting user experience sessions ensuring our design continues to be refined for existing and emerging workflows.

## DATA AVAILABILITY

All data, tools and documentation are available from Ensembl (https://www.ensembl.org), which has links to the REST API (https://rest.ensembl.org) and BioMart (https://www.ensembl.org/biomart/martview). There are no restrictions on data usage and all Ensembl code is on Github (https://www.github.com/Ensembl/) under an Apache 2.0 licence.

All queries about using Ensembl or Ensembl data or requests to host an Ensembl workshop can be addressed to our helpdesk (helpdesk@ensembl.org). We have a low traffic ‘Announce’ mailing list for updates and emails about new Ensembl releases, and a developers mailing list, to which anyone can subscribe (http://lists.ensembl.org/mailman/listinfo). We are also available via social media: Twitter (@ensembl), Facebook (Ensembl.org) and we maintain a blog at http://www.ensembl.info with updates for each new Ensembl release and other posts.

## References

[1] Ruffier M., Kähäri A., Komorowska M., Keenan S., Laird M.R., Longden I., Proctor G., Searle S., Staines D., Taylor K. Ensembl Core Software Resources: storage and programmatic access for DNA sequence and genome annotation. Database (Oxford). 2017; 2017:bax20. 10.1093/database/bax020 PMC5467575 28365736

[2] Yates A., Beal K., Keenan S., McLaren W., Pignatelli M., Ritchie G.R.S., Ruffier M., Taylor K., Vullo A., Flicek P. The Ensembl REST API: Ensembl data for any language. Bioinformatics. 2015; 31:143–145. 25236461 10.1093/bioinformatics/btu613 PMC4271150

[3] Rios D., McLaren W.M., Chen Y., Birney E., Stabenau A., Flicek P., Cunningham F. A database and API for variation, dense genotyping and resequencing data. BMC Bioinformatics. 2010; 11:238. 20459810 10.1186/1471-2105-11-238 PMC2882931

[4] Aken B.L., Ayling S., Barrell D., Clarke L., Curwen V., Fairley S., Fernandez Banet J., Billis K., García Girón C., Hourlier T. The Ensembl gene annotation system. Database (Oxford). 2016; 2016:baw093. 27337980 10.1093/database/baw093 PMC4919035

[5] Chen Y., Cunningham F., Rios D., McLaren W.M., Smith J., Pritchard B., Spudich G.M., Brent S., Kulesha E., Marin-Garcia P. Ensembl variation resources. BMC Genomics. 2010; 11:293. 20459805 10.1186/1471-2164-11-293 PMC2894800

[6] Zerbino D.R., Johnson N., Juetteman T., Sheppard D., Wilder S.P., Lavidas I., Nuhn M., Perry E., Raffaillac-Desfosses Q., Sobral D. Ensembl regulation resources. Database (Oxford). 2016; 2016:bav119. 26888907 10.1093/database/bav119 PMC4756621

[7] Herrero J., Muffato M., Beal K., Fitzgerald S., Gordon L., Pignatelli M., Vilella A.J., Searle S.M.J., Amode R., Brent S. Ensembl comparative genomics resources. Database (Oxford). 2016; 2016:bav096. 26896847 10.1093/database/bav096 PMC4761110

[8] Koepfli K.P., Paten B., Genome C.O.S., O’Brien S.J. The Genome 10K Project: a way forward. Annu. Rev. Anim. Biosci. 2015; 3:57–111. 25689317 10.1146/annurev-animal-090414-014900 PMC5837290

[9] Zhang G., Rahbek C., Graves G.R., Lei F., Jarvis E.D., Gilbert M.T. Genomics: bird sequencing project takes off. Nature. 2015; 522:34. 10.1038/522034d 26040883

[10] Teeling E.C., Vernes S.C., Dávalos L.M., Ray D.A., Gilbert M.T.P., Myers E., Bat1K C. Bat biology, genomes, and the Bat1K Project: To generate Chromosome-Level genomes for all living bat species. Annu. Rev. Anim. Biosci. 2018; 6:23–46. 29166127 10.1146/annurev-animal-022516-022811

[11] Lewin H.A., Robinson G.E., Kress W.J., Baker W.J., Coddington J., Crandall K.A., Durbin R., Edwards S.V., Forest F., Gilbert M.T.P. Earth BioGenome Project: Sequencing life for the future of life. Proc. Natl. Acad. Sci. U.S.A. 2018; 115:4325–4333. 29686065 10.1073/pnas.1720115115 PMC5924910

[12] Ota K.G., Kuraku S., Kuratani S. Hagfish embryology with reference to the evolution of the neural crest. Nature. 2007; 446:672–675. 17377535 10.1038/nature05633

[13] The UniProt Consortium UniProt: the universal protein knowledgebase. Nucleic Acids Res. 2017; 45:D158–D169. 27899622 10.1093/nar/gkw1099 PMC5210571

[14] O’Leary N.A., Wright M.W., Brister J.R., Ciufo S., Haddad D., McVeigh R., Rajput B., Robbertse B., Smith-White B., Ako-Adjei D. Reference sequence (RefSeq) database at NCBI: current status, taxonomic expansion, and functional annotation. Nucleic Acids Res. 2016; 44:D733–D745. 26553804 10.1093/nar/gkv1189 PMC4702849

[15] Kersey P.J., Allen J.E., Allot A., Barba M., Boddu S., Bolt B.J., Carvalho-Silva D., Christensen M., Davis P., Grabmueller C. Ensembl Genomes 2018: an integrated omics infrastructure for non-vertebrate species. Nucleic Acids Res. 2018; 46:D802–D808. 29092050 10.1093/nar/gkx1011 PMC5753204

[16] Li H. Tabix: fast retrieval of sequence features from generic TAB-delimited files. Bioinformatics. 2011; 27:718–719. 21208982 10.1093/bioinformatics/btq671 PMC3042176

[17] Lek M., Karczewski K.J., Minikel E.V., Samocha K.E., Banks E., Fennell T., O’Donnell-Luria A.H., Ware J.S., Hill A.J., Cummings B.B. Analysis of protein-coding genetic variation in 60,706 humans. Nature. 2016; 536:285–291. 27535533 10.1038/nature19057 PMC5018207

[18] Whirl-Carrillo M., McDonagh E.M., Hebert J.M., Gong L., Sangkuhl K., Thorn C.F., Altman R.B., Klein T.E. Pharmacogenomics knowledge for personalized medicine. Clin Pharmacol. Ther. 2012; 92:414–417. 22992668 10.1038/clpt.2012.96 PMC3660037

[19] Bickhart D.M., Rosen B.D., Koren S., Sayre B.L., Hastie A.R., Chan S., Lee J., Lam E.T., Liachko I., Sullivan S.T. Single-molecule sequencing and chromatin conformation capture enable de novo reference assembly of the domestic goat genome. Nat. Genet. 2017; 49:643–650. 28263316 10.1038/ng.3802 PMC5909822

[20] McLaren W., Gil L., Hunt S.E., Riat H.S., Ritchie G.R.S., Thormann A., Flicek P., Cunningham F. The Ensembl variant effect predictor. Genome Biol. 2016; 17:122. 27268795 10.1186/s13059-016-0974-4 PMC4893825

[21] Kumar P., Henikoff S., Ng P.C. Predicting the effects of coding non-synonymous variants on protein function using the SIFT algorithm. Nat. Protoc. 2009; 4:1073–1081. 19561590 10.1038/nprot.2009.86

[22] Alberto F.J., Boyer F., Orozco-terWengel P., Streeter I., Servin B., de Villemereuil P., Benjelloun B., Librado P., Biscarini F., Colli L. Convergent genomic signatures of domestication in sheep and goats. Nat Commun. 2018; 9:813. 29511174 10.1038/s41467-018-03206-y PMC5840369

[23] Zerbino D.R., Wilder S.P., Johnson N., Juettemann T., Flicek P.R. The Ensembl regulatory build. Genome Biology. 2015; 16:56. 25887522 10.1186/s13059-015-0621-5 PMC4407537

[24] Jolma A., Kivioja T., Toivonen J., Cheng L., Wei G., Enge M., Taipale M., Vaquerizas J.M., Yan J., Sillanpää M.J. Multiplexed massively parallel SELEX for characterization of human transcription factor binding specificities. Genome Res. 2010; 20:861–873. 20378718 10.1101/gr.100552.109 PMC2877582

[25] Jolma A., Yin Y., Nitta K.R., Dave K., Popov A., Taipale M., Enge M., Kivioja T., Morgunova E., Taipale J. DNA-dependent formation of transcription factor pairs alters their binding specificity. Nature. 2015; 527:384–388. 26550823 10.1038/nature15518

[26] Nitta K.R., Jolma A., Yin Y., Morgunova E., Kivioja T., Akhtar J., Hens K., Toivonen J., Deplancke B., Furlong E.E. Conservation of transcription factor binding specificities across 600 million years of bilateria evolution. Elife. 2015; 4:e04837. 10.7554/eLife.04837 PMC4362205 25779349

[27] Vaquerizas J.M., Kummerfeld S.K., Teichmann S.A., Luscombe N.M. A census of human transcription factors: function, expression and evolution. Nat. Rev. Genet. 2009; 10:252–263. 19274049 10.1038/nrg2538

[28] Traynelis J., Silk M., Wang Q., Berkovic S.F., Liu L., Ascher D.B., Balding D.J., Petrovski S. Optimizing genomic medicine in epilepsy through a gene-customized approach to missense variant interpretation. Genome Res. 2017; 27:1715–1729. 28864458 10.1101/gr.226589.117 PMC5630035

[29] Ioannidis N.M., Rothstein J.H., Pejaver V., Middha S., McDonnell S.K., Baheti S., Musolf A., Li Q., Holzinger E., Karyadi D. REVEL: An ensemble method for predicting the pathogenicity of rare missense variants. Am. J. Hum. Genet. 2016; 99:877–885. 27666373 10.1016/j.ajhg.2016.08.016 PMC5065685

[30] Kircher M., Witten D.M., Jain P., O’Roak B.J., Cooper G.M., Shendure J. A general framework for estimating the relative pathogenicity of human genetic variants. Nat. Genet. 2014; 46:310–315. 24487276 10.1038/ng.2892 PMC3992975

[31] Dong C., Wei P., Jian X., Gibbs R., Boerwinkle E., Wang K., Liu X. Comparison and integration of deleteriousness prediction methods for nonsynonymous SNVs in whole exome sequencing studies. Hum. Mol. Genet. 2015; 24:2125–2137. 25552646 10.1093/hmg/ddu733 PMC4375422

[32] Reva B., Antipin Y., Sander C. Predicting the functional impact of protein mutations: application to cancer genomics. Nucleic Acids Res. 2011; 39:e118. 21727090 10.1093/nar/gkr407 PMC3177186

[33] Adzhubei I., Jordan D.M., Sunyaev S.R. Predicting functional effect of human missense mutations using PolyPhen-2. Curr. Protoc. Hum. Genet. 2013; doi:10.1002/0471142905.hg0720s76. 10.1002/0471142905.hg0720s76 PMC4480630 23315928

[34] Liu X., Wu C., Li C., Boerwinkle E. dbNSFP v3.0: a one-stop database of functional predictions and annotations for human nonsynonymous and Splice-Site SNVs. Hum. Mutat. 2016; 37:235–241. 26555599 10.1002/humu.22932 PMC4752381

[35] Severin J., Beal K., Vilella A.J., Fitzgerald S., Schuster M., Gordon L., Ureta-Vidal A., Flicek P., Herrero J. eHive: an artificial intelligence workflow system for genomic analysis. BMC Bioinformatics. 2010; 11:240. 20459813 10.1186/1471-2105-11-240 PMC2885371

[36] Croft D., O’Kelly G., Wu G., Haw R., Gillespie M., Matthews L., Caudy M., Garapati P., Gopinath G., Jassal B. Reactome: a database of reactions, pathways and biological processes. Nucleic Acids Res. 2011; 39:D691–D697. 21067998 10.1093/nar/gkq1018 PMC3013646

[37] Casper J., Zweig A.S., Villarreal C., Tyner C., Speir M.L., Rosenbloom K.R., Raney B.J., Lee C.M., Lee B.T., Karolchik D. The UCSC Genome Browser database: 2018 update. Nucleic Acids Res. 2018; 46:D762–D769. 29106570 10.1093/nar/gkx1020 PMC5753355

[38] Kinsella R.J., Kähäri A., Haider S., Zamora J., Proctor G., Spudich G., Almeida-King J., Staines D., Derwent P., Kerhornou A. Ensembl BioMarts: a hub for data retrieval across taxonomic space. Database (Oxford). 2011; 2011:bar030. 21785142 10.1093/database/bar030 PMC3170168

[39] Gentleman R.C., Carey V.J., Bates D.M., Bolstad B., Dettling M., Dudoit S., Ellis B., Gautier L., Ge Y., Gentry J. Bioconductor: open software development for computational biology and bioinformatics. Genome Biol. 2004; 5:R80. 15461798 10.1186/gb-2004-5-10-r80 PMC545600

[40] Raney B.J., Dreszer T.R., Barber G.P., Clawson H., Fujita P.A., Wang T., Nguyen N., Paten B., Zweig A.S., Karolchik D. Track data hubs enable visualization of user-defined genome-wide annotations on the UCSC Genome Browser. Bioinformatics. 2014; 30:1003–1005. 24227676 10.1093/bioinformatics/btt637 PMC3967101

[41] Kluyver T., Ragan-Kelley B., Pérez F., Granger B., Bussonnier M., Frederic J., Kelley K., Hamrick J., Grout J., Corlay S. Loizides F, Schmidt B Jupyter Notebooks – a publishing format for reproducible computational workflows. Positioning and Power in Academic Publishing: Players, Agents and Agendas. 2016; Amsterdam: IOS Press 87–90.

[42] Stunnenberg H.G. International Human Epigenome Consortium and Hirst, M. The International Human Epigenome Consortium: a blueprint for scientific collaboration and discovery. Cell. 2016; 167:1145–1149. 27863232 10.1016/j.cell.2016.11.007

[43] The FAANG Consertium Andersson L., Archibald A.L., Bottema C.D., Brauning R., Burgess S.C., Burt D.W., Casas E., Cheng H.H., Clarke L. Coordinated international action to accelerate genome-to-phenome with FAANG, the Functional Annotation of Animal Genomes project. Genome Biol. 2015; 16:57. 25854118 10.1186/s13059-015-0622-4 PMC4373242

